# Clinical Significance of Cys-C and hs-CRP in Coronary Heart Disease Patients Undergoing Percutaneous Coronary Intervention

**DOI:** 10.21470/1678-9741-2018-0171

**Published:** 2019

**Authors:** Zheng Tan, Li Li, Yi Ma, Xuebin Geng

**Affiliations:** 1 Second Department of Cardiovasology, Tangshan Gongren Hospital, Hebei, China.

**Keywords:** Coronary Disease, Percutaneous Coronary Intervention, Cystatin C, C-Reactive Protein

## Abstract

**Objective:**

To investigate the clinical significance of serum cystatin C (Cys-C) and high-sensitivity C-reactive protein (hs-CRP) in coronary heart disease (CHD) patients undergoing percutaneous coronary intervention (PCI).

**Methods:**

One hundred and twenty-eight CHD patients were divided into drug treatment (56 cases) and PCI treatment (72 cases) groups, receiving conventional drug treatment and PCI plus conventional drug treatment, respectively. At admission time and 4 weeks after treatment, the left ventricular ejection fraction (LVEF), left ventricular end diastolic diameter, and left ventricular end systolic diameter were measured. At admission time and 24h, 72h, 1 week, and 4 weeks after treatment, the serum levels of Cys-C and hs-CRP were determined.

**Results:**

After 4 weeks of treatment, LVEF in the PCI treatment group was significantly higher than that before treatment (*P*<0.01) and it was significantly higher than in the drug treatment group at the same time (*P*<0.01). Cys-C and hs-CRP level in the PCI treatment group were significantly higher than in the drug treatment group 72h and 1 week after treatment (*P*<0.05 or *P*<0.01), respectively, but they were significantly lower than in the drug treatment group 4 weeks after treatment (*P*<0.01). There were obvious interaction effects between grouping factor and time factor in Cys-C (F=3.62, *P*<0.05) and hs-CRP (F=17.85, *P*<0.01).

**Conclusion:**

Serum levels of Cys-C and hs-CRP are closely related to the heart function in CHD patients undergoing PCI, and they may be used for predicting the outcome of PCI.

**Table:** 

Abbreviations, acronyms & symbols	
BMI	= Body mass index
BUN	= Blood urea nitrogen
CAG	= Coronary angiography
CHD	= Coronary heart disease
Cys-C	= Cystatin C
hs-CRP	= High-sensitivity C-reactive protein
ISR	= In-stent restenosis
LVEDD	= Left ventricular end diastolic diameter
LVEF	= Left ventricular ejection fraction
LVESD	= Left ventricular end systolic diameter
PCI	= Percutaneous coronary intervention
Scr	= Serum creatinine

## INTRODUCTION

Coronary heart disease (CHD) is the myocardial functional or organic lesion due to coronary artery stenosis or occlusion, or shortage of blood and oxygen supply^[1]^. It is one of the common causes of death due to cardiovascular diseases^[2]^. The formation of CHD is result of the interaction among multiple risk factors, in which the vascular endothelial inflammatory response caused by coronary atherosclerosis is an important mechanism^[3]^. Percutaneous coronary intervention (PCI) is one of the most important methods for the treatment of severe CHD, but in-stent restenosis (ISR) will affect the long-term curative effect^[4]^, and doctors and patients are very concerned about this problem. Selective coronary angiography (CAG) is the gold standard exam for diagnosis of ISR after PCI^[5]^. However, CAG is an invasive examination method and it is not conducive to the popularization of follow-up after PCI and timely finding ISR^[6]^. Seeking blood biochemical indicators for predicting ISR in clinics has become a focus in academic and research fields.

It is reported that some indicators in serum, in addition to the traditional risk factors (*e.g.*, age, gender, blood pressure, blood lipids, etc.), can also be used as independent risk factors for CHD and that they are closely related to the incidence of cardiovascular events^[7,8]^. High-sensitivity C-reactive protein (hs-CRP) is one of the inflammatory markers, which is synthesized by the liver. It can be used for predicting the risk of cardiovascular diseases^[9]^. Cystatin C (Cys-C) is a low-molecular-weight (13 kDa) protein that is a member of the cysteine protease family and is produced by all nucleated cells. Cys-C is involved in a variety of inflammatory reactions. It is a sensitive indicator of the degree of renal arteriosclerosis^[10]^ and it is also used as an emerging biomarker in cardiovascular disease^[11]^. This study aimed to investigate the clinical significance of Cys-C and hs-CRP in CHD patients undergoing PCI to provide a reference for the application of Cys-C and hs-CRP to monitor PCI treatment in CHD patients.

## METHODS

### Patients

One hundred and twenty-eight patients diagnosed with unstable angina pectoris of CHD by CAG from September 2015 to March 2016 in our hospital were enrolled in this study. There were 68 males and 60 females. The patients' ages were 51-78 years (59.8±6.4 years). According to the treatment method, the patients were divided into PCI treatment group (72 cases) and drug treatment group (56 cases). The following cases were excluded: i) patients with severe disease in respiratory system, blood system, or immune system; ii) patients with infectious disease, malignant tumor, or severe liver or kidney dysfunction; iii) patients with severe cardiac insufficiency (left ventricular ejection fraction [LVEF] <35%) or heart valve disease; iv) patients with unclear language expression or mental disorders. This study was approved by the Ethics Committee of GRYY-LL-2015-28. Written informed consent was obtained from patients or their families.

### Treatment

After admission, the drug treatment group received conservative treatment using statins, nitrates, beta blockers, anti-platelet drugs, angiotensin-converting enzyme inhibitors, and other conventional drugs. The PCI treatment group was given PCI treatment plus conventional drug treatment. There was no significant difference in diuretics use between the two groups. PCI was performed according to the "Guidelines for PCI in China" promulgated by the Chinese Medical Association of Cardiovascular Disease in 2012^[12]^.

### Detection of Heart Function Indexes

At the admission time and 4 weeks after treatment, echocardiography was performed using the Vivid 7 Cardiac ultrasonic examination instrument (GE Healthcare, WI, USA), and the heart function indexes including LVEF, left ventricular end diastolic diameter (LVEDD), and left ventricular end systolic diameter (LVESD) were measured.

### Detection of Cys-C and hs-CRP

At the admission time and 24h, 72h, 1 week, and 4 weeks after treatment, 5 ml of fasting venous blood were taken from the patients. After centrifugation at 2000 r/m (4ºC) for 5 min, the serum was obtained, and it was stored at -80ºC for use. Cys-C level was measured by particle-enhanced nephelometric immunoassay using BA800 automatic biochemical analyzer (Beijing Leadman Company, Beijing, China) and Cys-C kits (Shanghai Sangon Biological Engineering Technology and Service Co., Ltd., Shanghai, China). The level of hs-CRP was measured by immunofluorescence assay using i-CHROMA^TM^ immune fluorescence analyzer (Boditech Med Inc., Seoul, South Korea) and hs-CRP kits (Shanghai Sangon Biological Engineering Technology and Service Co., Ltd., Shanghai, China).

### Statistical Analysis

All statistical analyses were carried out using SPSS17.0 software (SPSS Inc., Chicago, IL, USA). Data were presented as mean ± standard deviation. Comparisons of enumeration data and measurement data were performed with χ^2^ test and t-test, respectively. Before and after treatment data were compared by paired-sample t-test. *P*<0.05 was considered as statistically significant.

## RESULTS

### Patients' General Information

The general information of the patients in the two groups is shown in Table 1. There is no significant difference of age, body mass index (BMI), serum creatinine (Scr) and blood urea nitrogen (BUN), smoking, CHD family history, hypertension prevalence rate, hyperlipidemia prevalence, or diabetes prevalence rate between the two groups (*P*>0.05).

**Table 1:** General information of patients in the two groups.

Group	Age (years)	BMI (kg/m^2^)	Scr (µmol /L)	BUN (mmol /L)	Smoking [n (%)]	CHD family history [n (%)]	Hypertension [n (%)]	Hyperlipidemia [n (%)]	Diabetes [n (%)]
Drug treatment (n=56)	58.2±5.2	22.2±2.6	55.6±8.4	5.9±1.1	13 (23.2)	12 (21.4)	14 (25.0)	12 (21.4)	10 (17.8)
PCI treatment (n=72)	60.9±5.3	22.4±2.2	57.2±9.3	5.8±1.5	18 (25.0)	16 (22.2)	17 (23.1)	15 (20.8)	13 (18.1)
t/x^2^	0.473	0.725	0.741	0.570	0.831	0.846	0.422	1.213	0.105
*P*	>0.05	>0.05	>0.05	>0.05	>0.05	>0.05	>0.05	>0.05	>0.05

BMI=body mass index; BUN=blood urea nitrogen; CHD=coronary heart disease; PCI=percutaneous coronary intervention; Scr=serum creatinine

### Comparison of Heart Function Indexes Before and After Treatment in the Two Groups

In the drug treatment group, LVEF, LVEDD, and LVESD before treatment were 52.12±6.18%, 48.95±6.78 mm, and 38.06±5.76 mm, respectively, and 1 month after treatment those were 58.46±7.84%, 49.21±5.46 mm, and 37.46±5.13 mm, respectively.

In the PCI treatment group, LVEF, LVEDD, and LVESD before treatment were 53.35±5 and 49.78±6.03 mm, and 37.53±4.97 mm respectively, and 1 month after treatment those were 69.60±8.21%, 51.03±5.24 mm, and 37.02±5.04 mm, respectively.

Before treatment, there was no significant difference between each index of the two groups (*P*>0.05). After treatment, LVEF in the PCI treatment group was significantly higher than that before treatment (*P*<0.01) and it was significantly higher than in the drug treatment group at the same time (*P*<0.01). LVEDD and LVESD had no significant difference before and after treatment in each group, with no significant difference between the two groups after treatment (*P*>0.05) (Table 2).

**Table 2:** Comparison of heart function indexes before and after treatment in the two groups.

Group	Heart function index	Before treatment	4 weeks after treatment
Drug treatment	LVEF (%)	52.12±6.18	58.46±7.84
	LVEDD (mm)	48.95±6.78	49.21±5.46
	LVESD (mm)	38.06±5.76	37.46±5.13
PCI treatment	LVEF (%)	53.35±5.46	69.60±8.21^ab^
	LVEDD (mm)	49.78±6.03	51.03±5.24
	LVESD (mm)	37.53±4.97	37.02±5.04

a *P*<0.01 compared with before treatment;

b *P*<0.01 compared with drug treatment group.

LVEDD=left ventricular end diastolic diameter; LVEF=left ventricular ejection fraction; LVESD=left ventricular end systolic diameter; PCI=percutaneous coronary intervention

### Comparison of Cys-C Level Before and After Treatment in the Two Groups

In the drug treatment group, Cys-C level before treatment and 24h, 72h, 1 week, and 4 weeks after treatment was 1.25±0.31, 1.29±0.34, 1.38±0.33, 1.26±0.34, and 1.24±0.32 mg/L, respectively, and in the PCI treatment group, Cys-C level was 1.32±0.35, 1.43±0.46, 1.58±0.51, 1.41±0.42, and 1.08±0.29 mg/L, respectively. There was no significant difference of Cys-C level between the two groups before treatment and 24h after treatment (*P*>0.05). Cys-C level in the PCI treatment group was significantly higher than in the drug treatment group 72h and 1 week after treatment (*P*<0.05 or *P*<0.01), respectively, but it was significantly lower than in the drug treatment group 4 weeks after treatment (*P*<0.01). The difference was also significant among different time points in each group (*P*<0.05 or *P*<0.01). Cys-C level in the PCI treatment group reached the peak at 72h after treatment and then decreased gradually. There was an obvious interaction effect between grouping factor and time factor (F=3.62, *P*<0.05) (Table 3).

**Table 3:** Comparison of Cys-C level before and after treatment in the two groups (mg/L).

Group	n	Before treatment	After treatment				F	*P*
			24 h	72 h	1 week	4 weeks		
Drug treatment	56	1.25±0.31	1.29±0.34	1.38±0.33	1.26±0.34	1.24±0.32	3.18	0.04
PCI treatment	72	1.32±0.35	1.43±0.46	1.58±0.51	1.41±0.42	1.08±0.29	8.65	<0.01
T		1.18	1.91	2.01	2.17	2.96	3.62	0.02
*P*		0.24	0.06	0.04	0.03	<0.01		

Cys-C=cystatin C; PCI=percutaneous coronary intervention

### Comparison of hs-CRP Level Before and After Treatment in the Two Groups

As shown in Table 4, in the drug treatment group, hs-CRP level before treatment and 24h, 72h, 1 week, and 4 weeks after treatment was 3.69±1.09, 4.05±2.01, 9.65±4.12, 7.04±3.24, and 3.85±1.02 mg/L, respectively, and in the PCI treatment group, hs-CRP level was 3.72±1.12, 4.13±1.98, 14.10±5.97, 9.76±5.41, and 2.35±0.97 mg/L, respectively. There was a significant difference among different time points in each group (*P*<0.05 or *P*<0.01). There was no significant difference of hs-CRP level between the two groups before treatment and 24h after treatment (*P*>0.05). In the PCI treatment group, hs-CRP level was significantly higher than in the drug treatment group 72h and 1 week after treatment (*P*<0.05 or *P*<0.01), respectively, but it was significantly lower than in the drug treatment group 4 weeks after treatment (*P*<0.01). In the PCI treatment group, hs-CRP level reached the peak at 72h after treatment and then decreased gradually. There was also an obvious interaction effect between grouping factor and time factor (F=17.85, *P*<0.01) (Table 4).

**Table 4:** Comparison of hs-CRP level before and after treatment in the two groups (mg/L).

Group	n	Before treatment	After treatment				F	*P*
			24 h	72 h	1 week	4 weeks		
Drug treatment	56	3.69±1.09	4.05±2.01	9.65±4.12	7.04±3.24	3.85±1.02	20.60	<0.01
PCI treatment	72	3.72±1.12	4.13±1.98	14.10±5.97	9.76±5.41	2.35±0.97	34.56	<0.01
T		0.15	0.23	4.76	3.33	8.49	17.85	<0.01
*P*		0.88	0.82	<0.01	<0.01	<0.01		

hs-CRP=high-sensitivity C-reactive protein; PCI=percutaneous coronary intervention

## DISCUSSION

PCI is a non-surgical method used to treat narrowed coronary arteries that supply the heart muscle with blood^[13]^. PCI has been clinically applied for almost 30 years and has become one of the main treatments for CHD^[14]^. PCI with coronary stent implantation has been demonstrated to consistently reduce the symptoms of coronary artery disease and decrease cardiac ischemia^[15]^. In this study, PCI plus conventional drug treatment was performed in CHD patients. Results showed that, after 4 weeks of treatment, LVEF in the PCI treatment group was significantly higher than that before treatment (*P*<0.01) and it was significantly higher than in the drug treatment group at the same time (*P*<0.01). This indicates that PCI can significantly elevate LVEF in CHD patients, improving the heart functions.

Cys-C is a low-molecular-weight secretory protein of the human body^[16]^. As an endogenous cysteine protease, it is an ideal indicator to reflect the glomerular filtration rate^[17]^. It is reported that there is a certain degree of correlation between the degrees of renal arteriosclerosis and coronary artery atherosclerosis^[18]^. Cys-C participates in the remodeling of extracellular matrix in CHD. In coronary atherosclerosis, the balance of Cys-C and its inhibitor is destroyed, leading to increased degradation in the internal elastic membrane of coronary artery and extracellular matrix. So, many monocytes and macrophages enter the intima, leading to coronary artery plaque formation^[19]^. Results of this study show that there was a significant difference of Cys-C level among different time points in each group (*P*<0.05 or *P*<0.01). Cys-C level in the PCI treatment group reached the peak at 72h after treatment and then decreased gradually. Cys-C level in the PCI treatment group was significantly higher than the in the drug treatment group 72h and 1 week after treatment (*P*<0.05 or *P*<0.01), respectively, but it was significantly lower than in the drug treatment group 4 weeks after treatment (*P*<0.01). The reason may be that the PCI results in the acute injury of the coronary artery and the application of contrast agent cause renal dysfunction. After 4 weeks, the renal function is completely recovered, so the Cys-C level is significantly decreased. This is similar to the results of previous clinical reports^[20,21]^.

hs-CRP is a non-specific inflammatory marker, which mainly reflects the human body injury, inflammation, and infection^[3]^. In recent years, with the application of ultra-sensitive detection technology, the trace amount of hs-CRP can be easily detected^[22]^. It is reported that hs-CRP mediated inflammation plays a key role in the occurrence and development of coronary atherosclerosis. The level of hs-CRP increases in the early stage of coronary artery lesion and a large number of hs-CRP can be detected in the early stage of coronary atherosclerotic plaque^[23]^. Results of this study showed that there was a significant difference of hs-CRP level among different time points in each group (*P*<0.05 or *P*<0.01). In the PCI treatment group, hs-CRP level reached the peak at 72h after treatment and then decreased gradually. In the PCI treatment group, hs-CRP level was significantly higher than the in the drug treatment group 72h and 1 week after treatment (*P*<0.05 or *P*<0.01), respectively, but it was significantly lower than in the drug treatment group 4 weeks after treatment (*P*<0.01). The reasons may be the PCI results in the acute injury of the coronary artery, leading to acute elevation of hs-CRP in the short term. After 4 weeks of operative recovery and coronary revascularization, the coronary endothelial function is more significantly restored. Therefore, the varieties of inflammatory factors are inhibited, and the level of hs-CRP also decreases significantly.

## CONCLUSION

In conclusion, the serum levels of Cys-C and hs-CRP are closely related to the heart function in CHD patients undergoing PCI and they may be used for predicting the outcome of PCI. This study still has some limitations. Firstly, the sample size of patients is relatively small, which may affect the persuasiveness of the results; secondly, troponin and creatine kinase are important factors for evaluating the outcome of CHD, however, they were not investigated in this study. These are the inadequacies of this study. In our next studies, the sample size should be further increased and other factors, including troponin and creatine kinase, should be investigated in order to obtain more satisfactory outcomes.

**Table:** 

Authors' roles & responsibilities	
ZT	Agreement to be accountable for all aspects of the work in ensuring that questions related to the accuracy or integrity of any part of the work are appropriately investigated and resolved; final approval of the version to be published
LL	Agreement to be accountable for all aspects of the work in ensuring that questions related to the accuracy or integrity of any part of the work are appropriately investigated and resolved; final approval of the version to be published
YM	Agreement to be accountable for all aspects of the work in ensuring that questions related to the accuracy or integrity of any part of the work are appropriately investigated and resolved; final approval of the version to be published
XG	Agreement to be accountable for all aspects of the work in ensuring that questions related to the accuracy or integrity of any part of the work are appropriately investigated and resolved; final approval of the version to be published
